# Expression of Osteoprotegrin and Osteoclast Level in Chronic Apical Periodontitis Induced with East Java Propolis Extract

**DOI:** 10.22037/iej.v13i1.18781

**Published:** 2018

**Authors:** Tamara Yuanita, Nanik Zubaidah, Sri Kunarti

**Affiliations:** a *Lecturer of Conservative Dentistry Department,* *Faculty of Dental Medicine, Universitas Airlangga, Surabaya, Indonesia*

**Keywords:** Chronic Apical Periodontitis, East Java Propolis, Intracanal Medicament

## Abstract

**Introduction:**

The objective of this animal study was to promote East Java propolis as a potential natural intracanal medicament for periapical chronic apical periodontitis bone resorption through evaluating the expression of osteoprotegrin (OPG) and osteoclast level.

**Methods and Materials:**

Propolis extract was produced using a maceration procedure. Thirty Wistar rats were divided into three groups. In group I, the control group, the first upper right molar constituted a healthy tooth. In group II, containing rodents with experimentally chronic

apical periodontitis, infection with *Enterococcus faecalis *ATCC29212 10^6^ CFU was performed. In group III, the treatment group, after being injected with *E faecalis*, 10 μL propolis was applied. It required 21 days to induce post-pulp chronic apical periodontitis infection. The rats were euthanised for immunohistochemical examination in order to measure the expression of OPG and to count histologically the number of osteoclast.

**Result:**

The expression of OPG and osteoclast constituted 17.5±1.58 and 6.4±0.96 in group I, 10±2 and 16.2±1.31 in group II and 17±1.69 and 7.5±1.08 in group III. Group I presented the highest level of OPG expression but the lowest level of osteoclast expression. There were significant differences between groups II and III and group I regarding OPG and osteoclast expression (*P*<0.05).

**Conclusion:**

East Java Propolis was a potential intracanal medicament promoting an increase in osteoprotegerin expression and a decrease in the number of osteoclasts thereby inhibiting osteoclastogenesis.

## Introduction

MPeriapical lesions, formed as a result of root canal infection, are accompanied by an immune response to the invading microbes and periapical bone destruction. Periapical lesions, also known as chronic apical periodontitis, represent an indicator of root canal treatment failure and often occur without presenting clinical symptoms. Radiographic images constitute the only means of diagnosing such lesions and the prevalence of periapical radiolucency is as high as 36% after dental root canal treatment has been performed. In cases of failure of such intervention, *Enterococcus faecalis (E. faecalis)* bacteria was found to be the most common causative agent [1, 2].

Chronic apical periodontitis occurs as result of a localized inflammatory response mediated by infiltrated inflammed cells and their products. The inflammatory process induces periapical bone resorption which constitutes a unique function of osteoclast with bony destruction representing another distinctive function [3, 4].

The receptor activator of nuclear factor kappa B (NF-*κB*) ligand (RANKL), receptor activator of NF-*κB* (RANK) and osteoprotegerin (OPG) all play crucial roles in regulating the differentiation, activation and survival of osteoclasts in both healthy and diseased tissues. RANKL and OPG work as competitive inhibitors to RANKL. RANK and RANKL interaction leads to bone destruction, whereas RANK and OPG interaction offers protection against it [5-7].

The main factors involved in osteoclast development begin with OPG, a potential inhibitor in osteoclastogenesis, which constitutes a member of the tumor necrosis factor (TNF) receptor superfamily. Molecular binding experiments confirmed that OPG associates with RANKL and functions as both a decoy receptor and dimer. RANK, like other members of the Tumor necrosis factor receptor (TNFR) superfamily, assembles into functional trimmers [8], while OPG is a natural inhibitor in periodontitis. Inhibition of RANKL is a promising new therapy for controlling bone loss. In human periodontitis, microorganisms initiate the activation of RANKL, the inhibition of which through the use of OPG markedly reduced alveolar bone loss in a mouse model of periodontitis [7-9].

Propolis, as a most commonly used herbal medicine in many countries of Asia, Europe, and America, demonstrates a broad spectrum of biological activity including; antibacterial, antibiofilm, antibiotics, antifungal, antioxidants, anti-inflammatory and anticancer [10-13]. Propolis is a natural source mainly having pharmaceutical applications. East Java propolis a resin mixture, yellow-brown in color, which is a product of the honey bee, *Apis mellifera [Zare Jahromi, 2012 #36]*. Propolis is collected from tree buds, sap, shrub or other plants and contains Caffeic Acid Phenethyl Ester (CAPE) (over 50%) as an active component [14].

This study aims to promote East Java propolis as a natural product promoting a decrease in periapical bone resorption in Wistar rats with induced chronic apical periodontitis. 

## Materials and Methods

This study used thirty, healthy, 12-week old, male Wistar rats, weighing between 130 and 150 grams which were divided into three groups (*n*=10), namely; a negative control group, an *E. faecalis* group and a group treated with East Java Propolis. Ethical clearance of the study protocol was done by the Health Research Ethics Committee of the Faculty of Dental Medicine, Universitas Airlangga No 28/KKEPK.FKG/III/2015. 

The extraction of propolis was conducted by means of maceration at the Balai Penelitian dan Konsultasi Industri, Surabaya, East Java. A total of 350 grams of raw East Java Propolis was macerated with 650 mL of 70% ethanol in a sealed container. The propolis and ethanol were agitated at 80 rpm. After 7 days, the maceration process was terminated, with the results being filtered. The maceration process was then repeated for 7 days until the color of the ethanol was considered to be stable. The solution was subsequently evaporated to produce an ethanol-free substance which was diluted with aquadest to obtain 12% propolis extract.

Wistar rats were anaesthetized with 80 mg/kg of *ketamine* (Ketalar, Warner Lambert, Ireland) and 10 mg/kg of *xylazine *(Xyla, Warner Lambert, Ireland) in sterile phosphat buffer saline (Darmstadt, Germany) by means of intraperitoneal technique. These rats were then attached to a retraction board and their right maxillary molar pulp opened using a low-speed electric handpiece (W&H, Zalsburg, Austria) with a round ¼ size bur (SS White burs Inc, Lakewood, NJ, USA). Group I, as the control group, possessed healthy teeth. Group II, as the *E. faecalis* group, had the exposed pulps immediately induced with 10 uL of *BHI-b* (Merck, Darmstadt, Germany) containing 10^6^ CFU of *E. faecalis* ATCC29212 bacteria, before being filled with glass ionomer cement (Fuji, GIC, Tokyo, Japan) to prevent contamination by oral micro-organisms. Meanwhile, the exposed pulps of group III, as the treatment group, were immediately induced with 10 uL *BHI-b* containing 10^6^ CFU of *E. faecalis* ATCC29212 bacteria. 10 µL of propolis was then applied and the cavity filled with GIC.

**Table 1:** The result of Tukey HSD test on osteoprotegrin.

	**Negative Control**	***E. faecalis***	**Propolis**
**Negative Control**	-	0.000 ^a^	0.804
***E. faecalis***	-	-	0.000 ^a^
**Propolis**	-	-	-

[a Significance (*P*<0.05)]

**Table 2:** The Mean (SD) of OPG and osteoclast expression for each group.

**Research Group (N=10)**	**Positive OPG**		**Osteoclast**	
	**Mean (SD)**	***P-*** **value**	**Mean (SD)**	***P-*** **value**
**Negative Control **	17.5 (1.58)	0.000 ^a^	6.4 (0.96)	0.000
***E. faecalis***	10 (2)		16.2 (1.31)	
**Propolis **	10 (1.69)		7.5 (1.08)	

[a Significance (*P*<0.05)]

**Figure 1:**
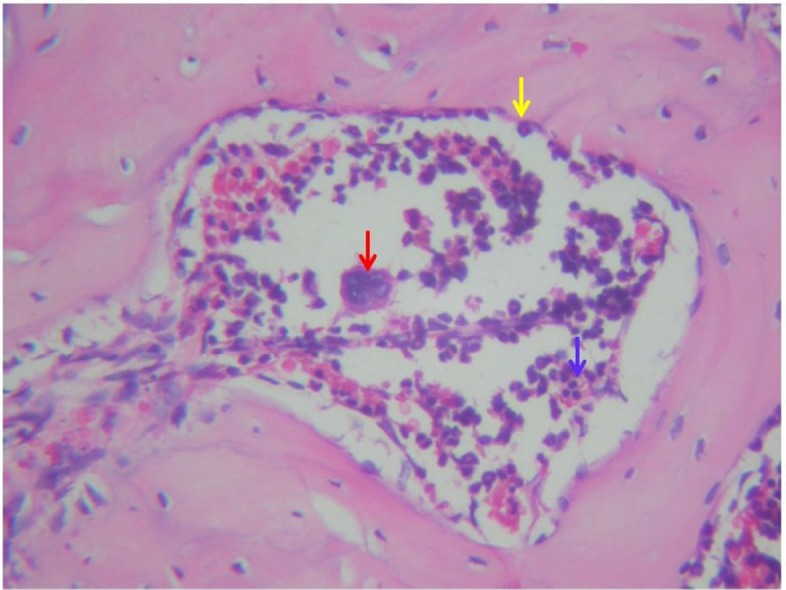
Number of osteoclast in periapical Wistar rats in propolis group. The red arrow indicates osteoclast, the yellow arrow: osteoblast and blue arrow: lymphocyte. (Magnification: 400×)

A period of twenty-one days was required to induce chronic apical periodontitis after pulp infection [15]. Wistar rats were sacrificed for immunohistochemical examination to enable measurement of the expression of OPG (Biotechnology, Santa Cruz, USA) under 400× magnification and histochemical examination with Hematoxillin Eosin also under 400× magnification to measure the osteoclast cells. Data was then collected and shown as a mean value±standard deviation. The differences between the groups were analyzed statistically by means of One-way ANOVA.

## Results

An ANOVA test was conducted to establish whether there were differences between the OPG expression and the number of osteoclasts within the groups after the application of East Java Propolis extract.

**Figure 2:**
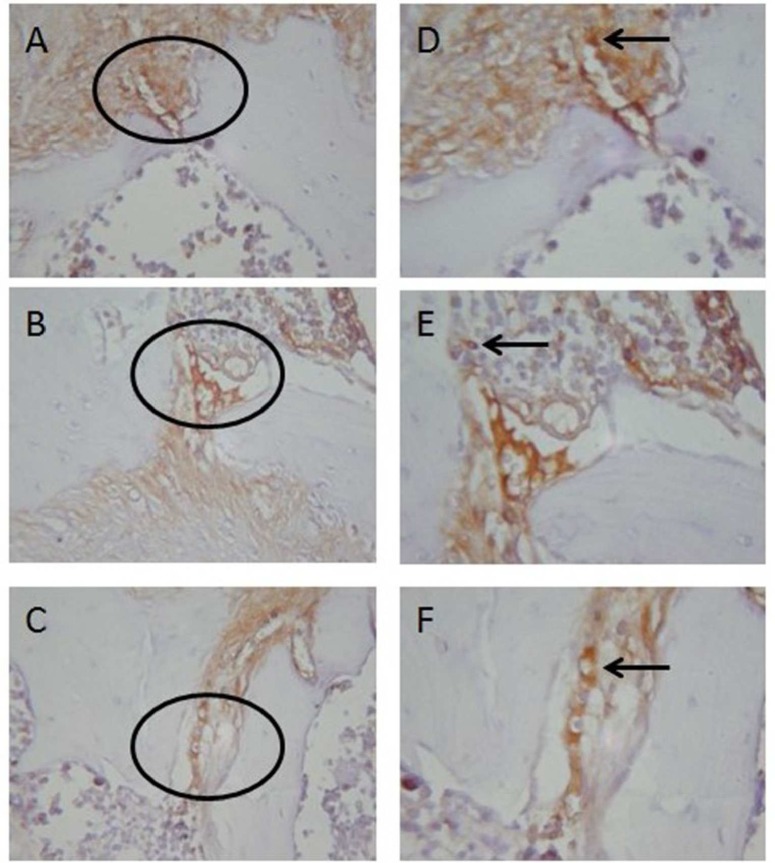
OPG Expression in periapical Wistar rats. *A)* The oval section represents a periapical area at 400× magnification in Control group; *B)* *E. faecalis *group; *C)* Propolis group; *D, E, F)* Under 1000× magnification then was conducted to confirm the oval area

Microscopic evaluation of OPG and osteoclast can be shown in Figure 1 and Figure 2. A Tukey HSD Test was conducted to determine the significance of OPG among the groups as shown in the Table 1. The results showed that there were significant differences in OPG between both the negative control group and the *E. faecalis* group within the propolis group. The significance of the differences in osteoclast between the groups was determined using a Tukey HSD Test as shown in the Table 2. The results showed that there were significant differences in osteoclast between both the negative control group and the *E. faecalis* group within the propolis group.

## Discussion

Bone is a dynamic tissue that undergoes continuous resorption and formation mediated by osteoclasts and osteoblasts. Bone remodeling, a prerequisite for normal life-long bone homeostasis, is regulated by a variety of factors such as cytokines, chemokines, hormones and biochemical stimulation. Osteoclasts are large, multinucleated cells which degrade the organic and inorganic matrices of bone and are fundamental to the pathogenesis of virtually all diseases associated with bone loss. Osteoclast is a significant cell which degrades bone [1, 5, 12].

Chronic periodontitis presents similar symptoms to apical periodontitis and occurs as a result of a local inflammatory response mediated by infiltrated inflammatory cells and their products. The inflammatory process leads to periapical bone resorption which is mediated by an unique osteoclast function [3, 9, 13].

*E. faecalis* is the most common causative bacteria in failed root canal therapy. Endodontic reinfection and periapical inflammation post-root canal treatment is caused, for the most part, by *E. faecalis *[16]. Its prevalence in repeated treatment through root canal therapy (RCT) is 89.6%, indicating that *E. faecalis* can survive even after RCT. Furthermore, this bacteria has become one of the recurrent causes of periapical disease. Molecular studies have confirmed *E. Faecalis *to be a common strain in teeth undergoing root canal treatment due to apical periodontitis lesions. The presence of *E. faecalis *reaches as high as 90% in apical periodontitis cases of RCT retreatment [17].

Indonesia is a country blessed with an abundance of natural resources. In the present day, the population of East Java increasingly employs herbal remedies as medication against several diseases. Such remedies became popular alternative treatments due to the desire to reduce the use of cheap and harmful chemical and synthetic substances in favour of natural materials. East Java propolis has a complex chemical composition facilitating its use as a herbal medicine [10-14].

Osteoclasts are multi-nucleated giant cells which can resorb bone matrix. Increasing numbers of osteoclasts lead to several metabolic bone-related diseases. In the oral cavity, chronic apical periodontitis is one common disease resulting from the increasing number of osteoclasts. OPG and RANK interaction leads to a reduction in the availability of RANKL to bind with RANK resulting in the inhibition of osteoclast differentiation [9]. The research reported here demonstrated [9]. The research reported here demonstrated that East Java propolis increases the expression of OPG. CAPE blocks the induction of NF-*κB* during RANKL induced *in vitro* osteoclast formation. CAPE has also been shown to significantly impede M-CSF and RANKL-induced osteoclast differentiation in a dose-dependent manner in *in* *vitro* mouse bone marrow-derived macrophages (BMMs) and the RANKL-induced osteoclast formation of mouse calvariae *in vivo*. They indicate that CAPE is a potential therapeutic agent in the inhibition of osteoclast bone resorption and can be employed to prevent bone loosening [11, 13].

The formation of multinucleated osteoclast is reduced due to an increase in OPG which is a natural decoy receptor effective in countering bone destruction by preventing the binding of RANKL with RANK [18]. Interestingly, it has been recently demonstrated that CAPE is an active component of honeybee propolisis negatively involved in osteoclastogenesis and bone resorption through an increase of OPG during osteoclast differentiation [14].

OPG is a soluble decoy receptor for RANKL that is a secreted member of the tumor necrosis factor receptor (TNFR) family that lacks a transmembran domain and is structurally distinct from RANK. OPG, produced by bone marrow stromal cells, inhibits osteoclast differentiation by binding RANK with high affinity, thereby preventing RANKL from binding to its cognate receptor, RANK. OPG interacts with RANKL and decreases its availability to bind RANK to the osteoclast precursors, thereby reducing the number of osteoclasts formed. OPG is also expressed by B-lymphocytes and DCs. Factors that regulate bone remodeling often do so by affecting the balance between RANKL and OPG synthesis. In diseases involving pathological bone remodeling, such as hypercalcemia of malignancy, this ratio has been shown to be abnormal [5, 8, 9].

The critical function of the osteoclast is to degrade organic and inorganic bone matrices. The accumulation of bone-degrading molecules on the resorption surface requires sustained physical contact between the osteoclast and the creation of a microenvironment functionally isolated from the general extracellular space. Osteoclasts accomplish this task by restructuring their actin cytoskeleton to form “a gasket-like” sealing zone which surrounds the resorptive milieu. The cell secretes HCl *via* an electrogenic H^+^ATPase (proton pump) and charge-coupled Cl^-^ channel thereby acidifying the resorptive microenvirontment. The bone’s organic matrix is demineralized and subsequently degraded by the lysosomal enzyme, cathepsin K. A combination of acidification and enzymatic digestion of inorganic and organic matrix components acts together to completely remove bone within this well-defined area. The inhibition of RANKL using OPG markedly reduced the alveolar bone lossin mouse model of chronic apical periodontitis. In fact, propolis increases OPG and protects RANKL binding to RANK [6, 19].

## Conclusion

East Java propolis is negatively involved in osteoclastogenesis by increasing OPG and the inhibition of multinucleated osteoclast formation.
